# Using Cellular Automata for Parking Recommendations in Smart Environments

**DOI:** 10.1371/journal.pone.0105973

**Published:** 2014-08-25

**Authors:** Gwo-Jiun Horng

**Affiliations:** Department of Computer Science and Information Engineering, Southern Taiwan University of Science and Technology, Tainan, Taiwan; University of Catania, Italy

## Abstract

In this work, we propose an innovative adaptive recommendation mechanism for smart parking. The cognitive RF module will transmit the vehicle location information and the parking space requirements to the parking congestion computing center (PCCC) when the driver must find a parking space. Moreover, for the parking spaces, we use a cellular automata (CA) model mechanism that can adjust to full and not full parking lot situations. Here, the PCCC can compute the nearest parking lot, the parking lot status and the current or opposite driving direction with the vehicle location information. By considering the driving direction, we can determine when the vehicles must turn around and thus reduce road congestion and speed up finding a parking space. The recommendation will be sent to the drivers through a wireless communication cognitive radio (CR) model after the computation and analysis by the PCCC. The current study evaluates the performance of this approach by conducting computer simulations. The simulation results show the strengths of the proposed smart parking mechanism in terms of avoiding increased congestion and decreasing the time to find a parking space.

## Introduction

A major research topic of intelligent transportation systems (ITSs) is the development of systems that will be capable of controlling the flow of vehicular traffic through crossroads, especially in urban environments [1]. ITS architecture provides a framework for the much needed overhaul of highway transportation infrastructure. The architecture's immediate effects include alleviating vehicle-traffic congestion and improving operational management to promote public safety via collision-avoidance improvements. Equipping vehicles with various onboard sensors and implementing vehicle-to-vehicle (V2V) communication will allow for large-scale sensing, decisions, and control actions in support of these goals [2].

Thus, innovative parking systems that meet near-term parking demands are needed. With wireless communication, computer, control, and electronic technologies, intelligent service-oriented parking management can improve parking space utilization and driver experiences while decreasing drivers' frustrations. The parking process can be a straightforward and non-stop process. From the management viewpoint, smart parking is an intelligent parking system. The parking process can be modeled as a birth-death stochastic process, and revenue can be predicted [5].

In urban areas, traffic congestion [6] is a major recurring problem in many countries due to the increased level of traffic, increased urbanization and the availability of cheaper vehicles. A wireless-enabled device called an onboard unit (OBU) allows a vehicle to set up a high-speed wireless link with road-side units (RSUs) using V2R communications. An RSU is strategically located at a critical position in the road systems. These OBUs and RSUs constitute a VANET that assumes the missions of traffic safety and intelligent transportation system control and management as well as supports any applications for the vehicles [7].

IEEE 802.11p radio technology is directly derived from IEEE 802.11a technology with some modifications for adapting to vehicular environments. This technology uses 75 MHz of the licensed spectrum, from 5.85 to 5.925 GHz, as part of the intelligent transportation system for dedicated short-range communications (DSRCs) in the USA [3]. The allocation of 75 MHz in the 5.9 GHz band that is licensed for DSRCs [4], which supports seven separate channels, could also enable the future delivery of rich multimedia content to vehicles within a short-to-medium range via either V2V or vehicle-to-roadside (V2R) links in VANETs.

To address the possible spectrum resource limitation in VANETs, cognitive radio has been considered as a potential solution. Cognitive radio (CR) networks have recently emerged as a promising technology for improving the utilization efficiency of the existing radio spectrum [8]. In a cognitive radio network, users opportunistically access the existing wireless spectrum without interfering with existing users.

This work contributes to the current literature via four objectives: 1) *To accomplish finding a parking space by using a cellular automata (CA) mechanism and a cognitive radio network model*; 2) *To reduce the time required to find a parking space and to avoid annoying the driver*; 3) *To raise the success rate, efficiency and stability of a parking space-finding process by a recommendation mechanism*; and 4) *To balance the temperature and reduce the CO_2_ on the main sections of roads.*

The remainder of this paper is organized as follows. Section 2 discusses the related literature. Section 3 describes the proposed mechanism and algorithm. Section 4 compares the proposed method with existing methods with reference to both analytical and simulation results. In Section 5, conclusions are presented, and recommendations are proposed for future research.

## Related Work

This section reviews important attempts to apply cognitive radio devices to vehicles based on V2R/R2V. Communication and segment information is from the CA model. We can obtain the segment information from conditions such as the local vehicle quantity, car location, and driving direction.

### A. V2R Communications

V2R communications enable vehicular networks to support a wide range of applications for enhancing the efficiency of road transportation. To support different ITS applications, both V2R communication and V2V communication must be supported in vehicular networks [9]. V2R communications allow vehicles to connect through their OBUs to the RSUs that belong to one or several service providers and download or upload various types of data that are related to a variety of applications. From the vehicles' perspective, due to the short duration that a vehicle spends at an RSU [10] [11] [12] [13], a vehicle commonly has only enough time to download a limited number of chunks or packets, e.g., those related to a single class of data. A vehicle can receive information on traffic conditions from an RSU through the road traffic management center. In such a network, decisions must be made on the opportunistic access of shared-use channels and the reservation of exclusive-use channels with respect to the vehicular nodes [14].

### B. Cellular Automata (CA)

The CA paradigm stems from researchers' attempts to model a seemingly complex process through a succession of simple local decision-making events. The paradigm's scientific and engineering implementation is based on the decomposition of a domain (a domain governed by physical laws) into a set of cells that form a uniform lattice. At the cell level, the decision-making process is implemented by rules that are functions of the neighboring sets of cells and the cells that belong to the set that is under consideration. In other words, the information is local by nature, and each cell in a given set acts as a single processor by receiving information from its neighborhood only. By applying the rules repetitively to locally updated physical quantities, the CA process converges into a description of the system's global behavior [15].

In the 1940s, Von Neumann proposed cellular automaton as a formal framework for studying the behavior of complex, extended systems. A cellular automaton is a discrete model of values in computability theory, mathematics, theoretical biology, and micro-structure modeling. A CA comprises a regular grid of cells, where each cell is in one of a finite number of states. The grid can be any finite number of dimensions, although the frequently studied CA model has one or two dimensions.

Time in the model is also discrete, and the state of each cell (Car) at time *t* depends on only the cell states in some well-defined neighborhoods of Car at time (*t*-1). These neighbors are a group of cells that are related to the specified cell, and they do not change. Every cell has the same rule for updating based on the values in the neighborhood [16].

**Figure 1:**
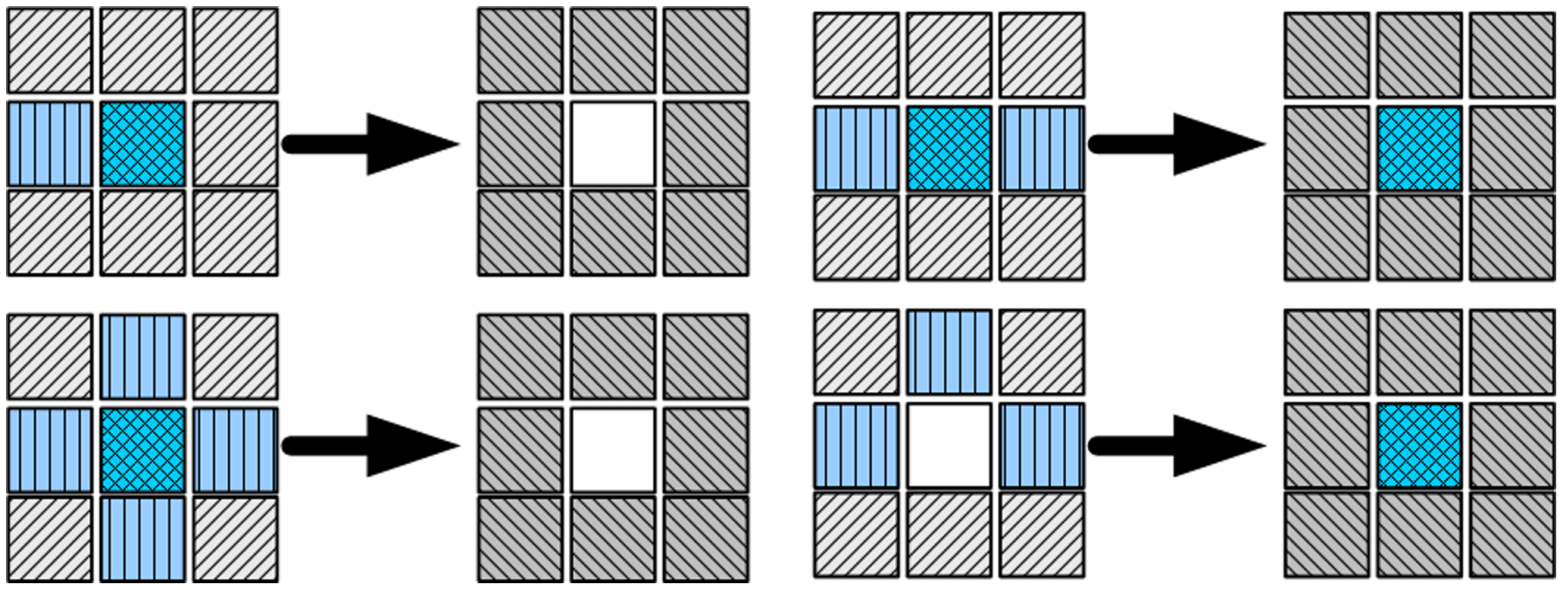
Rules for the “game of life”.

In the “game of life”, cells have two possible states (dead or alive), which depend on a Moore neighborhood that comprises the eight cells surrounding the given cell. The rules are called the “game of life” because they are usually explained by the assumption that a cell symbolizes some living organism that requires its neighbors to survive, as shown as Figure 1.

Cells with fewer than two neighbors die of loneliness.
Living cells with two neighbors stay alive.
Non-living cells with three neighbors become alive. Living cells stay alive.
Cells with more than three neighbors die of overcrowding.

### C. Cognitive Radio (CR)

CR is one of the most exciting advances of telecommunications over the past few years. The underlying goal of a CR system is to establish ubiquitous peer-to-peer connections by intelligently taking advantage of unused temporal and spatial spectrum resources of the users, as discussed in [7].

Cognitive radio is a special type of software-defined radio that can estimate the communication parameters and intelligently adapt itself to the changing environment. To achieve the desired objective (e.g., channel utilization), an intelligent decision-making algorithm is required for cognitive radio [17]. CR is suitable in V2R communications due to its highly mobile and dynamic networking environment such that spatial and temporal reuse of the licensed spectrum can be realized in a much easier and cheaper way compared with other types of wireless networks.

### D. Smart Parking

Caliskan et al. [18] propose a parking system in which parking automats are the producers of resource reports. Wired sensors [19], such as 1) inductive loops, 2) pneumatic road tubes, 3) magnetic sensors, 4) piezoelectric sensors, and 5) weigh-in-motion systems are widely used. Stiller et al. [20] propose cognitive automobiles that have the intelligence to handle some of the events that occur in real scenarios. Benson et al. [21] propose an RF transceiver and antenna with an ATMega 128 L micro-controller system, which applies image processing to detect the vehicles [22], [23]. Funck et al. [24] use images to detect the parking space. Caliskan et al. [25] propose a parking system in which parking automats are the producers of resource reports. The infrastructure uses IEEE 802.11 to broadcast these reports as raw text packets. Therefore, some wireless sensors can be applied to parking space detection.

## Methods

Parking is limited in almost every major city in the world and leads to traffic congestion, air pollution, and driver frustration [5]. While many cities have already presented intelligent urban traffic and recommendation mechanisms, drivers are concerned with the remaining parking spaces in the parking lot when urbanization attracts a high density of vehicles. Currently, many applications are implemented on navigation devices that can determine a vehicle's location, a nearby parking lot, and how far the lot is; this information helps the driver navigate to the destination. Nevertheless, when the vehicle arrives at the parking lot, it may have to wait for other vehicles that are leaving. This situation can annoy the drivers and is time consuming.

In this work, we propose an innovative adaptive recommendation mechanism for smart parking. The recognitive RF module will transmit the vehicle location information and parking space requirements to the parking congestion computing center (PCCC) when the driver must find a parking space. Moreover, we use the CA model mechanism for those parking spaces in the parking lot; this mechanism can adjust to the situation that some parking lots are full and some are not. Here, the PCCC can compute the nearest parking lot, the parking lot status and the current or opposite driving direction with the vehicle location information. We take the driving direction into consideration because road congestion can be reduced and finding a parking space can be sped up by avoiding backtracking.

The recommendation will be sent to the drivers through the wireless communication CR model after the computation and analysis by the PCCC. The recommendation will include the parking space location and will navigate the driver to the destination; the driver's directions to turn left, turn right, or go straight will also be included.

**Figure 2:**
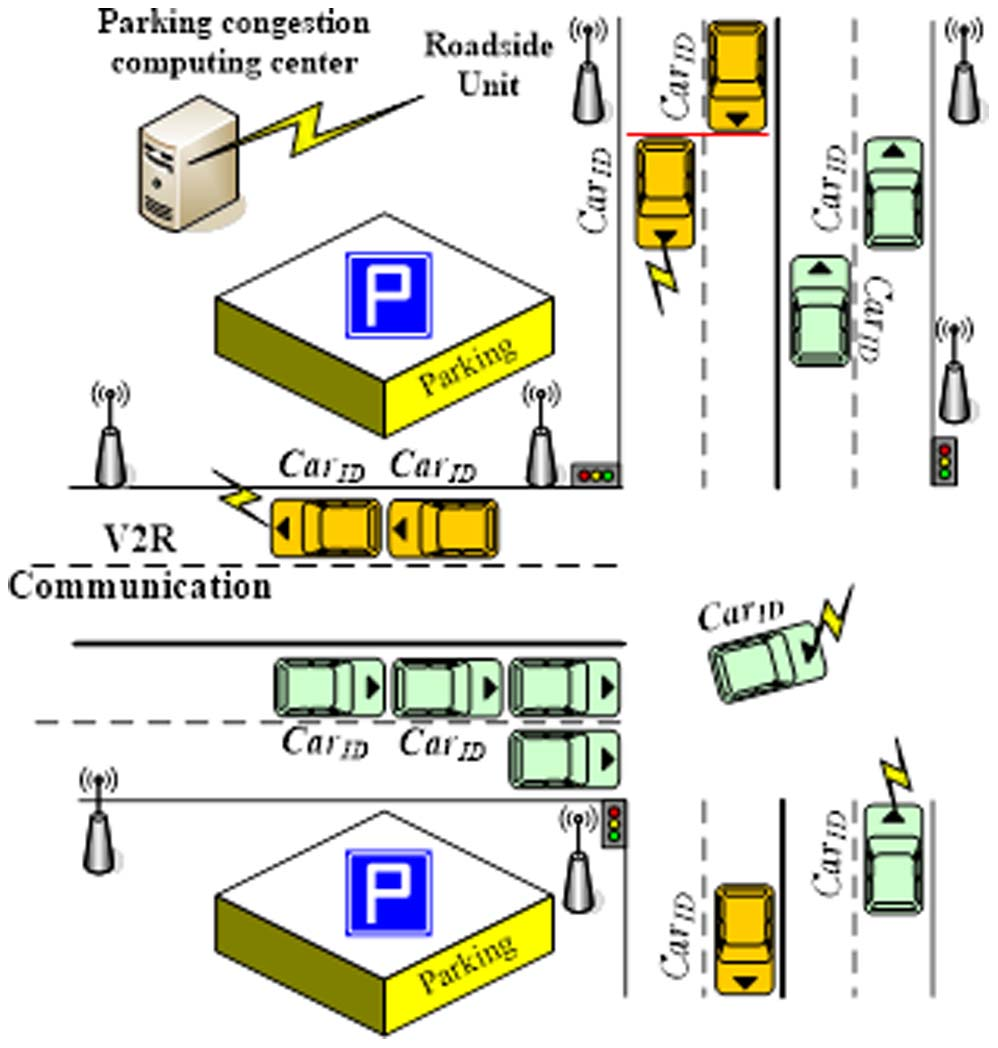
Car society scenario.

In this section, we present a car society scenario in an urban traffic environment. We have a limited communication range for the RSU infrastructures over every road segment, as shown in Figure 2. We consider that there is a PCCC in the urban area that can collect the parking space status of the parking spaces in each parking lot, the data packets of the message and the request from each vehicle. The PCCC will analyze this information and provide feedback to the drivers. We then propose a type of V2R communication model in which there is a parking space near the intersection and a roadside unit device that supports a certain communication area. The vehicle can utilize the CR model by using OBUs and wireless communication to transmit the *Car_ID_* vehicle status and parking requests to the PCCC when the vehicle is moving. The spectrum does not need to register the ID, and the PCCC will respond with the recommendations to the drivers.

The *Car_ID_* comprises the following categories of information: *CP*(*t*) denotes the current position information of the car; *d* denotes the destination information; the vehicle's navigation information in next time interval is Nav{*t*+1, *direction*[*n*]}, where *direction*[*n*] 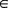 *direction*[*TurnLeft*], *direction*[*TurnRight*], and *direction*[*GoStraight*]; and *v_ID_* denotes the vehicle ID number. A CarID is composed of a cognitive signal module that denotes *Car_ID_*  =  *v_driver_*{*CP*(*t*), *d*, *Nav*[*t*+1, *direction*[*n*]]}+*v_ID_*.

**Figure 3:**
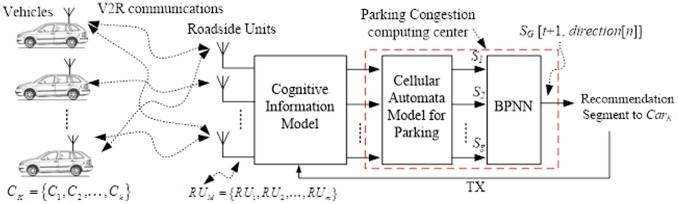
The system structure diagram.

We describe the system structure diagram in Figure 3. We denote each vehicle as *C_K_* = {*C_1_*, *C_2_*,…, *C_k_*}, where *K* 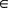 {1,2,…,*k*}; we denote RSU as *RU_M_* = {*RU_1_*, *RU_2_*,…, *RU_m_*}, where *M* 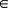 {1,2,…,*m*}. The communication type between the vehicles and RSUs is V2R. The information from the vehicles that can be received by the RSUs will need to be recognized and analyzed within the cognitive radio model. Environments that constitute vehicle societies are under study.

The cognitive radio system can dynamically access the available radio spectrum resources in an opportunistic fashion; thus, the spectrum utilization can be significantly improved, and the user service requirements can be better satisfied. We consider using the CA mechanism, which is defined for a wide range of CA models within a car society.

The information of *Car_ID_*(*CP*(*t*)) is received and analyzed by the PCCC for the road segment *S_G_*; hence, we define every road segment *S_G_* = {*S_1_*,*S_2_*,…,*S_g_*}, where *G* 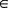 {1,2,…,*g*}; CA is suitable for discrete and dynamic situations.

This work uses a CA model with a cognitive radio network mechanism to compute the *Car_ID_* information. We then use a back-propagation neural network (BPNN) model to obtain the most adaptive result by inputting multivariate data near *S_G_* and the conditions of *Car_ID_*(*CP*(*t*)). The most adaptive result is the nearest *Car_ID_*(*CP*(*t*)), which has the same direction as *Car_ID_*(*d*). The recommendation information for *C_K_* is denoted as *Car_ID_  =  v_driver_* {*CP*(*t*), *d*, *Nav*[*t*+1, *direction*[*n*]], *S_G_* [*t*+1, *direction*[*n*]]}+*v_ID_*, which first considers the distance.

### A. CA Mechanism

CA is a mathematical model that follows continuous adaption rules to change the movement of the vehicles in the parking lot and the number of vehicles. While the parking lot is almost a local issue, the influencing field can be a whole urban area after a long-term evolution. Hence, in this work, the influencing field will expand by time evolution.

In the CA mechanism that we propose, the state machine is the power of evolution for vehicle movement in the parking lot and the number of vehicles. The state machine will travel in the vehicle moving direction at time *t*. Then, whole cells (vehicles) in every parking lot of the urban area will synchronously travel for a time period from *t* to *t*+1 through the state machine under a non-continuous time segment. Suppose that we use the CA mechanism to indicate that every parking lot can continuously accept vehicles parking in the area. This situation works only when the travel time, movement and number of vehicles are smooth. If the status and travel time cannot stay steady in the parking lot, then there will be congestion, i.e., dead cells appear in the CA mechanism. Thus, we propose a CA mechanism to attempt to maintain steady conditions in the parking lot and prevent congestion for a longer time.

The basic expression of a parking lot without a CA model is shown in equation (1). In equation (5), we assume that there are *K* vehicles and that the *P^th^* vehicle has similar location information as the *K^th^* vehicle.
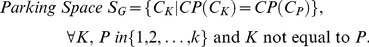
(1)


We assume that the state *Parking Space_G_*(*t*+1) at (*t*+1) only depends on the states of its neighborhood at time t. A parking lot update of a vehicle at any iteration is performed based on information from the neighborhood and the vehicle itself. The functional relation used to update the state of a parking lot is typically called the update rule, i.e.,
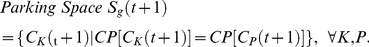
(2)


We assume that a vehicle belongs to a road segment; the number of vehicles at (*t*+1) depends on the stats of a given neighborhood at *t*, and *F* denotes the transfer function to the CA mechanism. This type of iteration model is represented by:
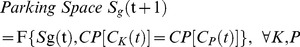
(3)


We then use CA to reduce the congestion in a parking lot. In an urban vehicular network mechanism, we have continuous moving vehicles in a real traffic environment. Thus, in the next time interval, vehicles in the parking lot can change their direction of motion to leave the parking lot, or we can have a new vehicle drive in. We propose this innovative CA mechanism to achieve our goal due to the active mode. According to our description of the notation, vehicles in the parking lot will synchronously change state in the next time interval by using the CA state machine rules.

### B. Cognitive Multichannel Mechanism

Vehicular communication devices on IEEE 802.11p are equipped with a CR to utilize the very-high-frequency (VHF)/UHF TV broadcast frequency bands when the 5.9 GHz licensed spectrum band is not available. The vehicles have a CR capacity from secondary users in the network [7].

Both radio (VHF, microwaves, and millimeter waves) and infrared waves have been used in experimental V2V systems. Infrared and millimeter waves allow communication only along lines of sight; 3 VHF and microwaves allow broadcast communications. VHF can provide long links but at low speeds; the spectrum used in the mainstream is the microwave spectrum.

**Figure 4:**
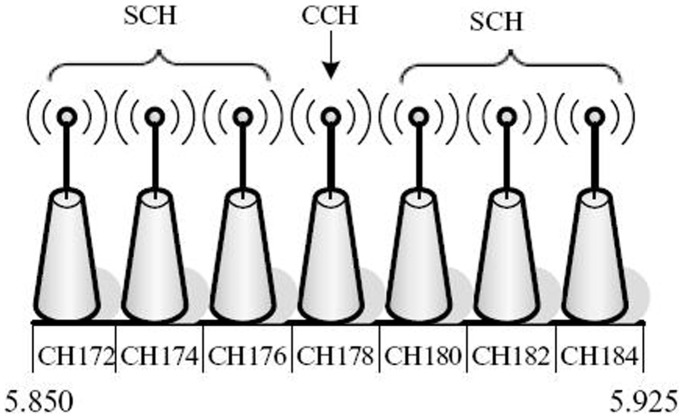
IEEE802.11p channel distribution [22].

We propose the items of *CAR_ID_* information by using a multi-channel model. In this work, we design the vehicle to be available to transmit the *CAR_ID_* information to the RSU by one of six interest channels. One channel is for inter-vehicle emergency communications, as shown in Figure 4. Let *CH_l_* = {*CH_1_*, *CH_2_*,…, *CH_7_*} denote multiple channels, and the respective multi-channel model is given by:
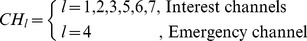
(4)


The U.S. Federal Communications Commission (FCC) has approved 75 MHz of bandwidth for the Intelligent Transportation System (ITS) at frequencies between 5.850 to 5.925 GHz, which is divided into seven channels [19]. As shown in Figure 4, one of the seven channels, CH178, is designated as the control channel (CCH); this channel is responsible for the safety of the relevant applications, including systems control and management with high priority. The other six channels are service channels (SCH), and they support non-safety-relevant applications [20]. The Wireless Access in Vehicular Environments (WAVE) system, which is being standardized according to IEEE P1609.4 [21] and IEEE 802.11p [19], has been widely accepted as the basis of IVC and RVC due to its ability to provide broadband and low-latency wireless communications over short-to-moderate distances [20].

Spectrum sensing in the secondary network is a key step in interference avoidance with the primary user network. Unlicensed users, also called cognitive users, must monitor the spectrum activities continuously to find a suitable spectrum band for possible utilization and to avoid possible interference with the licensed users, who are also called the primary users. The primary users have the priority for service, and the above spectrum sensing by cognitive users includes the detection of possible collisions when a primary user becomes active in the spectrum that is momentarily occupied by a cognitive user; a reallocation of the communication channels occurs [23].

In this work, we consider using the *CarID* recognition mechanism combined with the CR sensing model to transmit road information and smart parking information under a car society. Whether the mechanism is for the detection of temporal or spatial spectrum gaps, spectrum sensing in CR involves deciding whether the primary signal is present from the observed signals [2]. This approach can be formulated as the following two hypotheses:
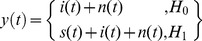
(5)where *y*(*t*) is the received signal at the CR user, *s*(*t*)s(t) is the primary signal, *i*(*t*) is the interference, and *n*(*t*) is the additive white Gaussian noise (AWGN). *H_0_* and *H_1_* denote the hypotheses that correspond to the absence or presence of the primary signal, respectively. Thus, from the observation *y*(*t*), the CR user must decide between *H_0_* and *H_1_*.

**Figure 5:**
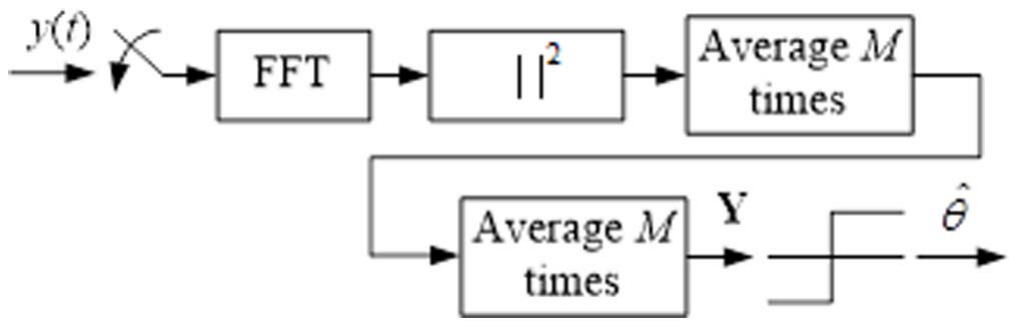
Schematic representation of the energy detector.

Energy detection [24] is the simplest spectrum sensing technique, which is shown in Figure 5. An energy detector simply treats the primary signal as noise and determines the presence or absence of the primary signal based on the energy of the observed signal. In this case, the simultaneous sensing of a number of sub-bands can be realized by simple scanning of the power spectral density of the received wide-band signal. The spectral component on each spectrum sub-band of interest is obtained from the fast Fourier transform of the sampled received signal. The test statistics of the energy detector are obtained as the observed energy summation within *M* consecutive segments.
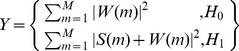
(6)where *S*(*m*)S(m) and *W*(*m*)W(m) denote the spectral components of the received primary signal and the white noise on the sub-band of interest in the *m*th segment, respectively. The decision of the energy detector regarding the sub-band of interest is given by:
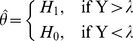
(7)where the threshold λ is chosen to satisfy a target false-alarm probability.

## Adaptive Parking Space Recommendation Mechanism

In this section, we consider analyzing the number of vehicles in the parking lot and the road segment information with the location information *Car_ID_*(*CP*(*t*)) of vehicle *C_K_*. We then compute the nearest parking lot for the vehicle and choose a smooth traffic route for the driver. Additionally, we will provide the next direction (i.e., go straight, turn left, or turn right). We propose to use the computational model of a back-propagation neural network (BPNN) to analyze the most adaptive recommendation for distributing the vehicles in each urban parking lot. Furthermore, this approach can avoid vehicles gathering in certain parking lots, which heats the roads and raises the CO_2_ density in an urban area.

In general, the traditional road-guide device calculates a complete routing path on the basis of the road information that is provided by the government. When the driver chooses a routing path that is calculated by the BPNN model, the driver must follow this chosen path. The driver cannot change this routing path immediately. In the PCCC, we consider a vehicle's current status and destination information. The PCCC can simulate any possible routing path (road segment).

Then, we assume that the adjustment factors are the congestion rating and the road distance. The PCCC will calculate and select the next suitable road segment from *S_G_*. When the PCCC calculates the results, the PCCC will send the next suitable road-segment direction message to the driver: *Car_ID_*  =  *v_driver_*{*CP*(*t*), *d*, *Nav*[*t*+1, *direction*[*n*]]}+*v_ID_*.

In this section, we use the CA model to analyze the vehicle quantity information of the road segments. We then compare the result with the location information *Car_ID_*(*CP*(*t*)) of the car *C_K_* for a recommended road segment. For the next step, we pass on directions to the car (i.e., go straight, turn left or turn right). To analyze the adaptive recommendation, we propose a BPNN model to avoid situations in which vehicles converge on specific road segments. This model is suitable for the entire stretch of a road in an urban area.

Neural networks train themselves by reviewing related data and patterns. A neural network's “learning” curve is much like a person's learning curve: the more data input there is, the faster and more substantive the learning. A neural network is very different from the characteristics of current computer learning. Neural networks reflect the human brain's operating mode, which calls for the organization of various network paradigms, especially back-propagation networks.

**Figure 6:**
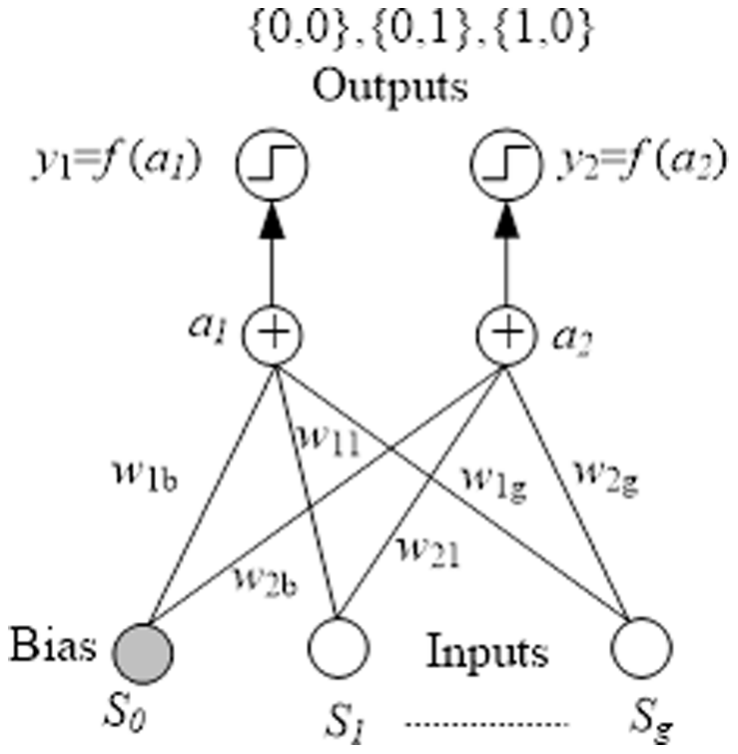
BPNN for segment recommendation.

In this work, we use the dynamics of a multiclass single-layer neural network during training. The learning algorithm is the BP model. In Figure 6, the single-layer neural network has {*S*_1_, *S*_2_,…, *S_g_*} external input items and one bias *S_0_* input, where *bias* denotes the threshold value. The *bias* is much like a weight, except that it has a constant input of 1; *W* denotes a set of synapses or connecting links, where each link is characterized by a weight. *W* and *bias* are the adjustable scalar parameters of the neurons. The adaptive segment recommendation model is given by:
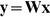
(8)

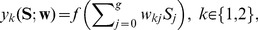
(9)where *f* is the activation function, and *y_k_* is the output item.

We assume that the given vehicle has three choices {*S*_1_, *S*_2_, *S*_3_} from the given current position to the destination position. We allow these choices to be the input of the BPNN; the TCCC will set two parameters that could influence the road conditions: the congestion rating and the road distance. First, the parameter bias and **W** are randomized by the TCCC. Then, we calculate the error function e through the feedback until we satisfactorily minimize the error function. These results can optimize the parameter **W**. The neural analysis model consists of {*S*_1_, *S*_2_, *S*_3_}, the congestion rating, road distance, parameter bias and parameter weight. We treat the step function's decision mechanism as the output. This study has two output items: *y*_1_ and *y*_2_. These output items, {*y*_1_, *y*_2_}, involve three models: {0, 0}, {0, 1}, and {1, 0}. We treat y1 as representative of left turns and *y*_2_ as representative of right turns; when *y*_1_ and *y*_2_ are equal to 0, the given vehicle should go straight. We provide the next appropriate driving directions at the next time slot in such a way that the vehicle can avoid congestion. To provide these recommendations, we use the R2V mechanism.

In this work, the goal is not only to make the spectrum reusable and to reduce the consumption but also to satisfy the requirement to easily find a parking space in the car society. We use a BPNN model to reach a recommendation mechanism requirement. Therefore, the innovative CA mechanism combines with the parking recommendation method to provide the following benefits: First, the mechanism sends a message to the driver based on the distance to the destination and direction. Second, it can avoid segment congestion in the urban areas. Third, the lack of a delay in finding a parking space will prevent the driver from feeling annoyed.

## Results and Discussions

In this section, we present the simulation results of a car society using the CA mechanism under a smart parking environment. Table 1 shows the simulation parameters and the range of values. The chosen parameters should resemble those of high network density urban areas.

**Table 1:** Simulation Parameters.

Factor	Range of values
Simulation area	5.0×5.0 km^2^
Number of vehicles	300–1800
Number of lanes	2 per direction
Number of parking spaces	200–1200
Distance between intersections	350 m
Distance between RSUs	500 m
Vehicle speed	10–70 km/h

In the current study, we assume that in a real-world vehicle network environment, a vehicle's speed is affected by the Doppler effect and by any data communication's success rate on the spectrum sensing problem. After we simulated and analyzed the different numbers of vehicles and the different vehicle speeds through Matlab, we found that the vehicles traveling at a high speed maintained a low success rate of spectrum sensing. Thus, we considered many factors, including the simulation area, number of vehicles, number of lanes, number of intersections, distance between intersections, RSUs, and adjustable vehicle speed. This paper evaluates the performance of the proposed scheme. This work randomly distributes 300 to 1800 vehicles in a 5.0×5.0 km^2^ field.

**Figure 7:**
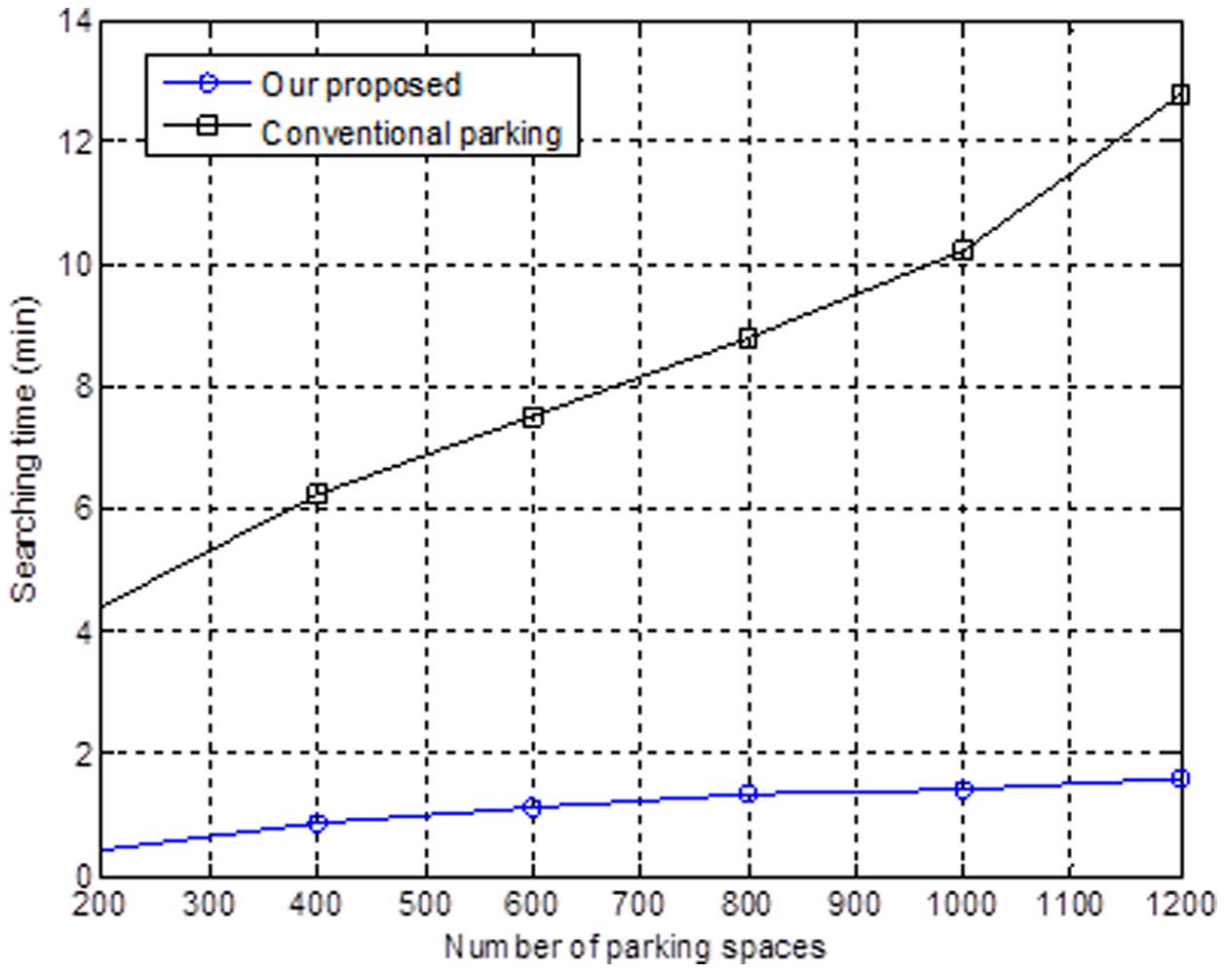
Parking space searching time.

Using a CA mechanism and cognitive radio network model, this work introduces a new mechanism of finding a parking space. The results can help a driver to find a parking space more quickly and reduce vehicle-traffic congestion. We then discuss the emulation result that the mechanism we propose is faster and more convenient than the conventional opportunism method. Moreover, this approach will not lead to nearby segment congestion, based on the low probability. Figure 7 shows the relationship between the number of parking spaces and the search time. We also show the same result with the conventional opportunism method (i.e., a shorter search time when there are fewer parking spaces). Suppose that we have many urban areas that have a very large number of vehicles, and the government will set up more parking spaces. While the government will set up fewer parking spaces when an urban area has a smaller number of vehicles, it is easier and faster to find a parking space.

Another reason for the emulation result is that drivers will spend more time finding preferred parking spaces and consequently have a longer search time. On the other hand, drivers will intuitively follow the recommendations to park their vehicles using our mechanism.

**Figure 8:**
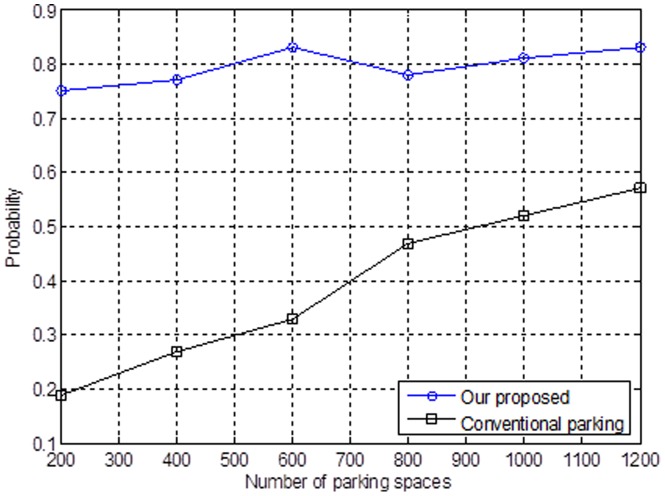
Relationship between the number of parking spaces and the probability.

Suppose that we have a larger number of vehicles and parking spaces in the urban area. Figure 8 indicates that the available parking spaces will decrease when the parking spaces of the parking lot are used progressively. The main reasons that the likelihood of finding a parking space decreases by the conventional opportunism method are listed as follows: 1) the range of parking fees; 2) the different locations of the properties; and 3) whether it is convenient.

However, having fewer parking spaces will indirectly influence nearby road-traffic congestion situations. Hence, the mechanism that we propose will build up a PCCC includes the overall number of parking spaces in the urban area. Each vehicle will receive the recommended parking lot and the parking space by the cognitive radio model. By doing so, we can distribute the parking spaces and avoid competing for specific parking spaces.

**Figure 9:**
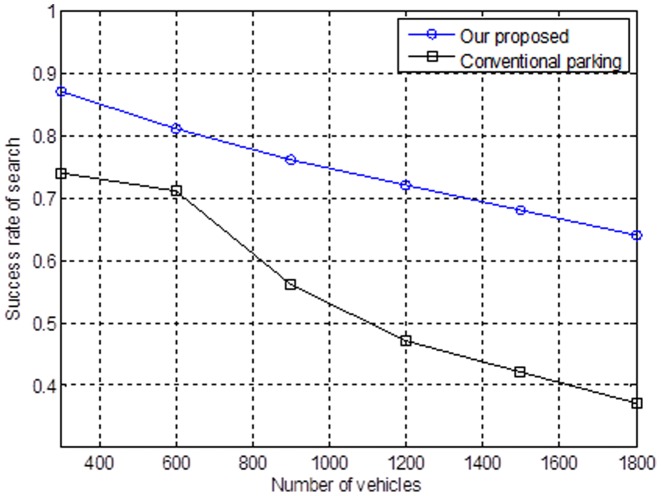
Relationship between the number of vehicles and the success rate of the search.

Suppose that we have a larger number of vehicles in the downtown area of a city. We assume that there is an increasing number of vehicles and vehicles are finding parking spaces. Figure 9 shows the mechanism of finding a parking space; we propose the success ratio will slightly decrease. For the conventional opportunism method, the success ratio of finding a parking space will significantly decline.

**Figure 10:**
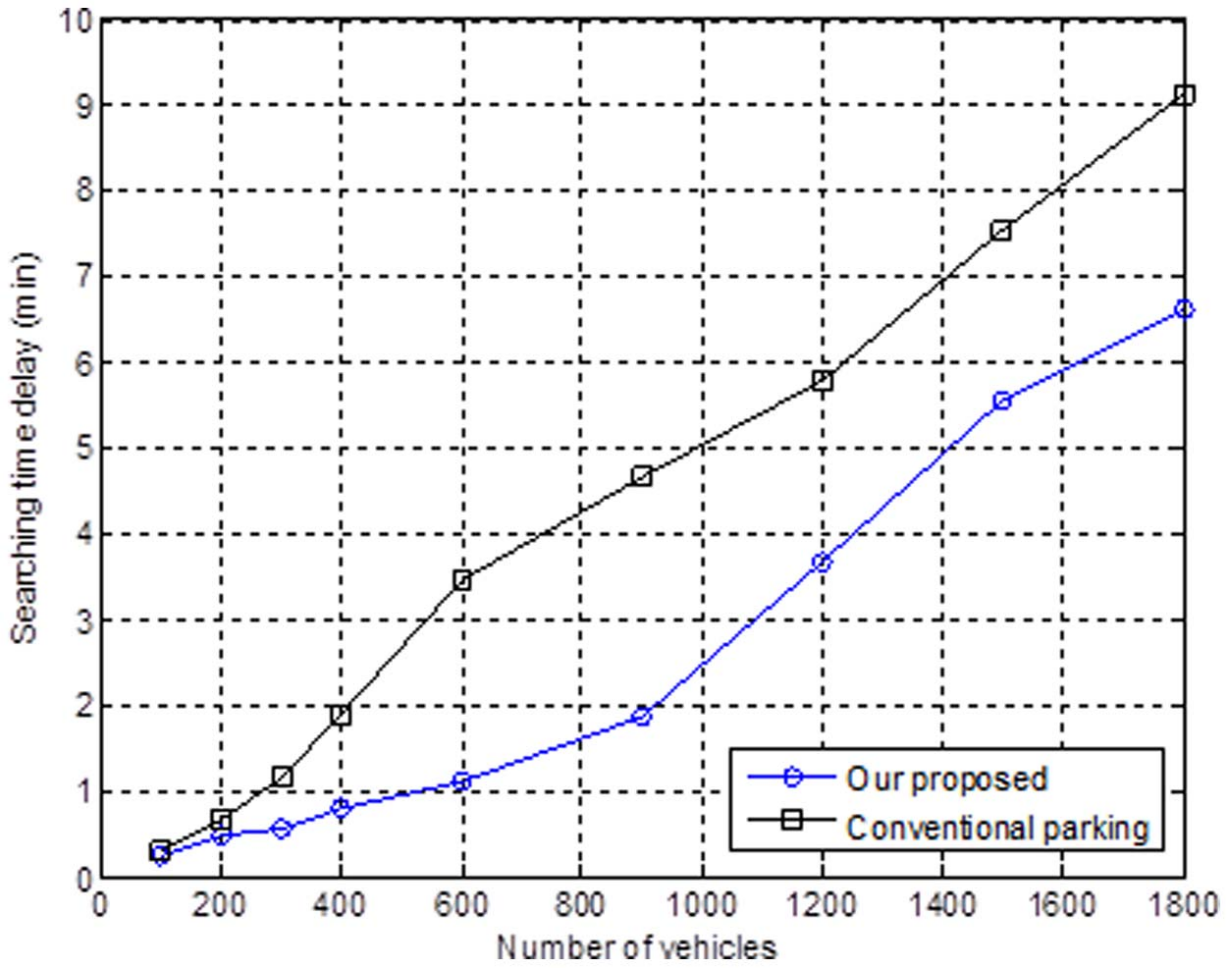
Relationship between the number of vehicles and the search time delay.

We then discuss the relationship between the number of vehicles and the search time delay. As shown in Figure 10, one of the main reasons that there is a higher delay from the search time is the rising number of vehicles. Moreover, the conventional opportunism method allows the vehicle to park as soon as the driver finds a parking space or by the driver's preference. The situation will very quickly impact the delay due to the search time. We propose that the innovative mechanism will provide an adaptive recommendation for the nearest parking space. This mechanism can avoid the segment congestion issue and can control the delay search time in a progressively increasing situation, even when the number of vehicles increases sharply and the road segment has a heavy flow of cars.

**Figure 11:**
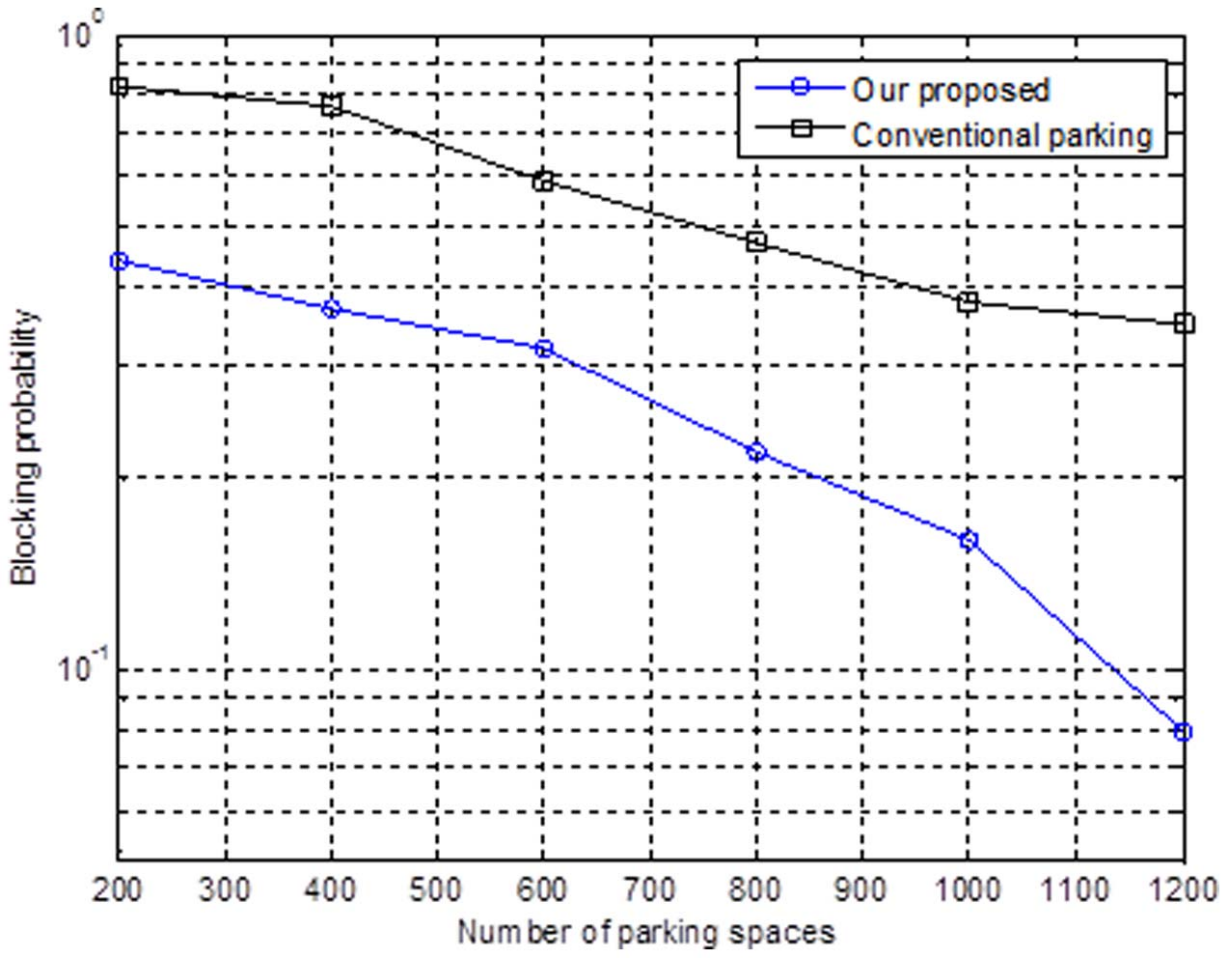
Relationship between the number of parking spaces and the blocking probability.

In Figure 11, we discuss the issue that the driver will have a delay in finding a parking space when the parking lot is becoming full. This situation will also lead to traffic congestion. Moreover, consider an environment that is downtown and has more vehicles and parking spaces. We can easily imagine that the blocking rate will increase when there is a decrease in the number of parking spaces. In other words, when fewer vehicles park in the parking lot, there will be a lower blocking ratio. In this work, the mechanism with which we provide recommendations to the drivers for parking their vehicles is also much better than parking by the opportunistic method.

**Figure 12:**
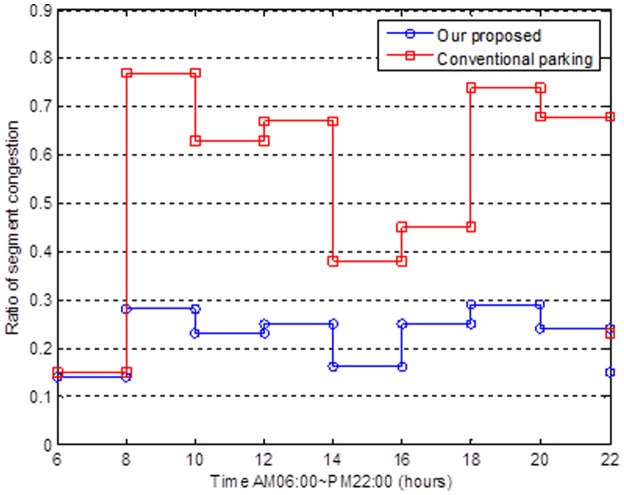
Relationship between the time and ratio of the segment congestions.

Figure 12 shows the relationship between the time (AM06∼PM22) and the ratio of the segment congestion; we emulate different time segments from six AM to ten PM (i.e., the rush hour and off-peak traffic time). Note that the ratio of the segment congestion is high during the rush hour and above average during the off-peak traffic time. The ratio of the segment congestion will have a sharp drop until the afternoon or after ten PM. We can obtain a low, stable segment congestion ratio by analyzing the situation under the conditions that we set, while there is a high correlation between finding a parking space and the segment congestion. In this work, we propose that the novel mechanism can avoid the high segment congestion and more equally separate the flow of cars.

The algorithm dynamically coordinates the agents' visiting behavior. In other words, the theme park problem is a dynamic coordination problem that requires the coordination of many individual behaviors and the optimization of individual or social satisfaction using distributed information [26]. In another study [27], the theme park problem involved the adjustment of the visitors' schedules to reduce congestion.

**Figure 13:**
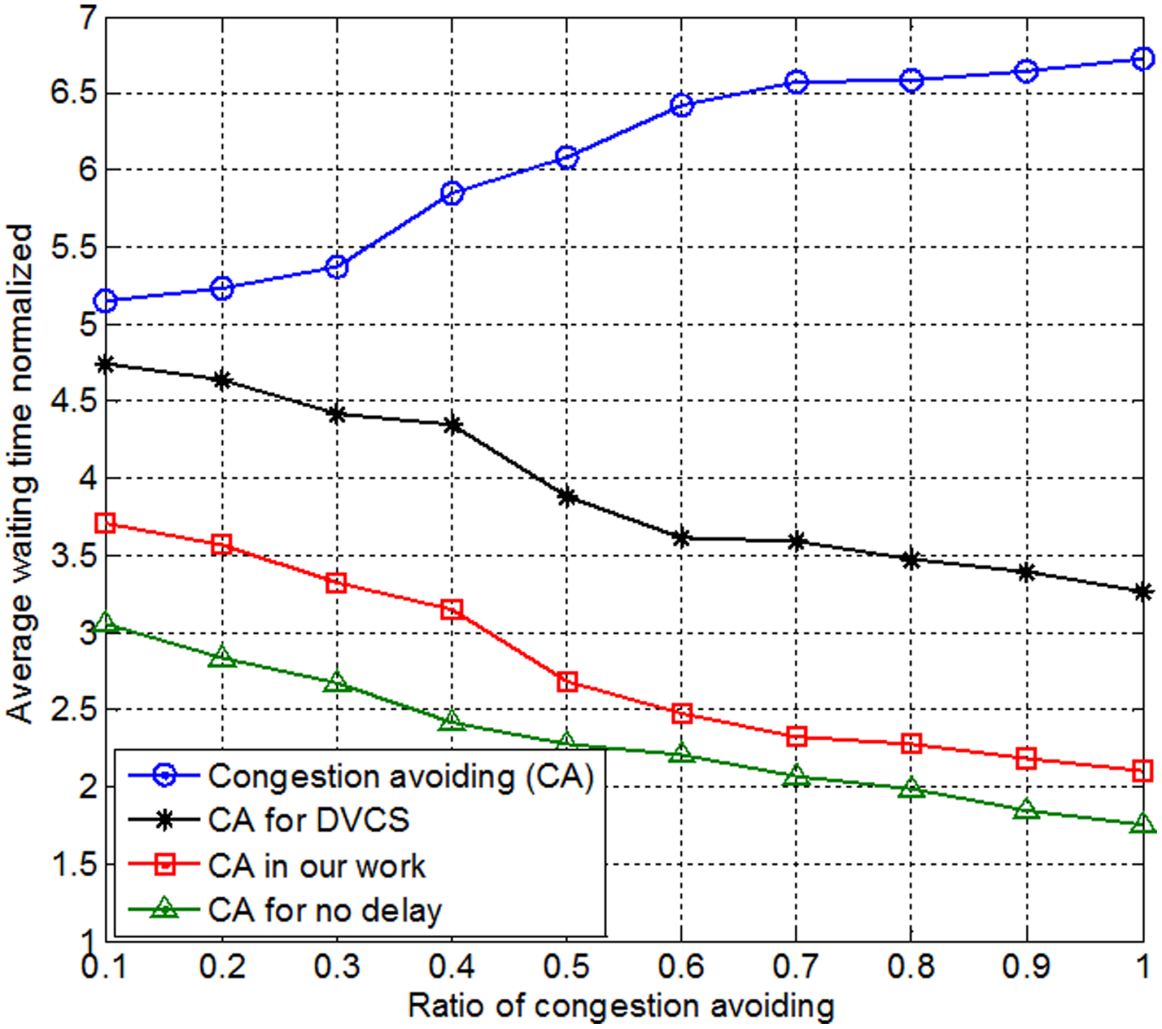
Analysis of congestion avoidance ratio.

Figure 13 shows the two factors in the analysis results; one was the normalized average wait time, and the other was the ratio of number of congestion to the number of arrivals. The ratio of congestion avoidance ranged from 0.1 to 1 and was smaller than the other two mechanisms. In other words, cars that used our proposed mechanism to choose a path had a smaller average wait time than tour groups that used another mechanism. Therefore, our mechanism enhanced the system's performance and the parking quality.

Congestion avoidance (CA) means that each car can immediately into the urban area with the fewest visitors after having visited their scheduled current urban; there is no time delay problem. This type of CA situation is unachievable, but we used it for the purposes of comparison. CA means that the system server calculates the wait time from the queue length at each scene at that time. Each agent avoids congestion by referring to the estimated wait time. That is, the server provides each agent with the estimated wait time at each attraction as congestion information. Each agent can register its next destination and stand in an exclusive queue.

Figure 13 presents an efficiency analysis of the congestion avoidance ratio and the average wait time. Our proposed mechanism had smaller wait times than the other two mechanisms, which indicates that our proposed mechanism was associated with an enhancement of the system efficiency and the parking quality. CA increased gradually because it suffered from the time delay problem.

**Figure 14:**
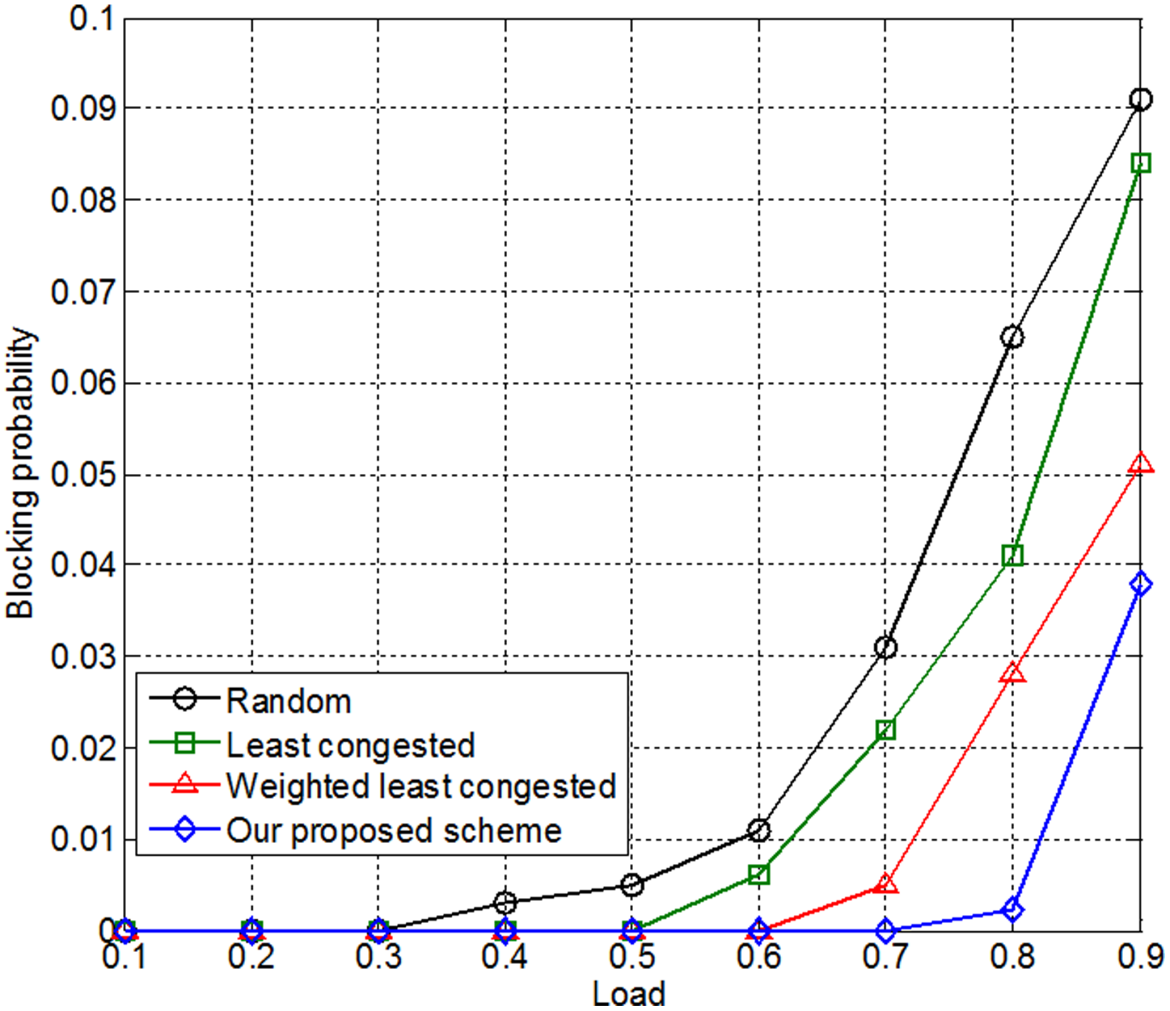
Relationship between the traffic load and the blocking probability.

Figure 14 shows the relationships between traffic load and blocking probability according to four methods: random, least congested, weighted least congested, and our proposed method. The simulation results show that the blocking probability of the random model was very high when the traffic load was 0.8, and the blocking probability of the least congested model was slightly less. The blocking probability of our proposed method was less than that of the weighted least congested scenario when the traffic load was 0.8.

**Figure 15:**
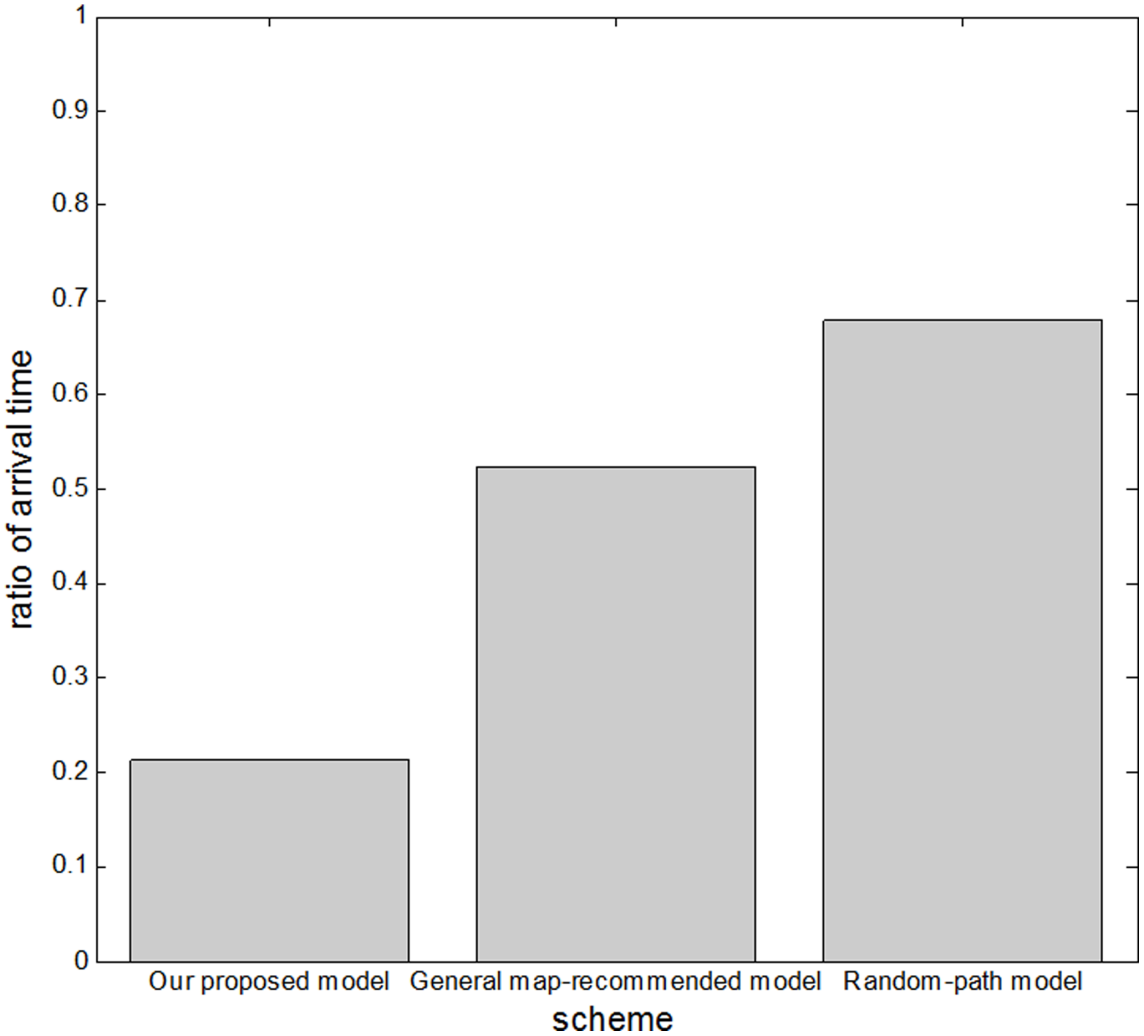
Relationship between the three models and the ratio of arrival time.

Because we consider many different environment and sites, we compare the ratio of arrival time based on our proposed mechanism, the general recommended mechanism and the random path mechanism. Figure 15 shows our analysis of the three modes of sightseeing relative to the ratio of arrival time. Our proposed mechanism resulted in an earlier arrival time than the other models. In other words, the time a tour group would need to complete a path using our proposed model is less than the time needed when using one of the other models. Thus, our proposed mechanism outperforms the others in terms of the ratio of arrival time.

## Conclusions

This work proposes a new mechanism for finding a parking space using a CA model and a cognitive radio network model. This system can help a driver find a parking space more quickly and thereby reduce traffic congestion. We then discuss the emulation result that the mechanism we propose is faster and more convenient than the conventional opportunistic method. Moreover, based on the low probability, this new approach will not lead to nearby segment congestion. The simulation results reveal the effectiveness of the method in terms of its higher levels of stability, searching time delay, congestion avoidance, the success rate of the search and the ratio of the segment congestion that typically characterize conventional parking and opportunistic parking. We conclude that our proposed protocol achieves substantial improvements for smart parking in a traffic network in terms of avoiding congestion and reducing the time to find a parking space.
